# Cortical Venting: A Simple Surgical Adjunct for the Treatment of Long Bone Nonunion

**DOI:** 10.7759/cureus.70193

**Published:** 2024-09-25

**Authors:** Sean M Wade, Jordan G Tropf, Robert M Putko, Jean-Claude G D'Alleyrand

**Affiliations:** 1 Department of Orthopedic Surgery, Captain James A. Lovell Federal Health Care Center, North Chicago, USA; 2 Department of Surgery, Uniformed Services University of the Health Sciences, Bethesda, USA; 3 Department of Orthopedic Surgery, United States Naval Hospital Guam, Agana Heights, USA; 4 Department of Orthopedic Surgery, Naval Medical Center San Diego, San Diego, USA; 5 Department of Orthopedic Surgery, University of Pennsylvania, Philadelphia, USA

**Keywords:** cortical venting, exchange nailing, long bone fractures, nonunion, orthopaedic trauma

## Abstract

Nonunions are a vexing problem for the orthopedic surgeon. Herein, we describe an adjunct to the standard exchange nailing procedure adapted from an established limb lengthening technique in which cortical vents are drilled adjacent to the nonunion site. These transcortical drill tunnels facilitate local dispersion of the osteogenic intramedullary reamings around the nonunion site during the exchange nailing, whereby the extruded reamings serve as autograft for the nonunion. This simple adjunctive technique can increase the likelihood of achieving union when performed with an exchange nailing procedure as demonstrated by our case series of recalcitrant tibia and femoral nonunions successfully treated when this surgical adjunct was implemented.

## Introduction

Long bone nonunion is a debilitating condition associated with devastating functional, psychological, and financial ramifications [1-3]. Factors contributing to its development include mechanical instability, inadequate bone contact, infection, a compromised soft tissue envelope, and deficient vascularity at the fracture site. A nonunion is characterized as aseptic or septic, hypertrophic, oligotrophic, or atrophic, which is determined by its etiology. Successful treatment is contingent upon addressing the underlying factors contributing to its formation.

Tibial and femoral shaft nonunions typically present with severe pain and inability to bear weight on the affected extremity. Nonunion of the tibia is more common than nonunion of any other long bone fracture with a rate of occurrence ranging from 5% to 15% [1,4,5]. Femoral shaft nonunion occurs less frequently, but still affects 0.9%-4% of patients [6-9]. The goal of treatment for this condition is to achieve union and regain pain-free function of the extremity. Treatments for this condition focus on increasing mechanical stability and improving biological conditions around the challenged bone.

Multiple treatment options exist for addressing tibial and femoral nonunion encompassing both non-operative and operative methods. Treatment options include the use of external bone stimulators, open application of bone graft material to the nonunion, augmentative plating across the nonunion, Ilizarov ringed external fixation, nail dynamization, and exchange nailing. Although an in-depth review of these treatment options is beyond the scope of this article, we will describe exchange nailing because it is a well-established and commonly implemented technique for treating long bone nonunions that we have modified to further encourage successful healing of this condition. In a standard exchange nailing operation, the original intramedullary nail is removed, the canal is over-reamed by 1 mm, and then a new nail 2 mm larger than the original is inserted in the canal [10]. The larger diameter nail imparts greater rigidity and increases its endosteal contact than the original nail, thereby improving the mechanical stability of the construct [10-12]. Moreover, the biological effects from reaming of the intramedullary canal increase periosteal blood flow in response to the disruption of endosteal circulation, which further promotes bone healing at the nonunion site [13]. Despite the high rates of union associated with the standard exchange nailing procedure [11,12,14] and the multitude of other established treatment options, persistent long bone nonunions continue to pose a problem for orthopedic surgeons [15]. Herein, we describe an adjunctive technique to the standard exchange nailing procedure for treating tibial and femoral shaft nonunions adapted from an established limb lengthening technique to increase the likelihood of achieving union [16,17]. This modification involves drilling cortical vents adjacent to the nonunion to facilitate the egress of osteogenic intramedullary reamings around the nonunion site. We demonstrate this adjunctive technique’s effectiveness in a limited consecutive series of patients with recalcitrant nonunions of the tibia and femur who then went on to achieve union after this technique was implemented as part of their nonunion treatment.

Technique description

Patient positioning for the exchange nailing is dependent on the surgeon's preferred position for addressing the nonunited bone. For tibial nonunions, the patient is positioned supine on the operating room table with the operative leg placed in either the semi-extended position or flexed at the knee. For femoral nonunions, the patient is positioned supine or in the lateral decubitus position with or without a fracture table, depending on the surgeon’s preference for performing an exchange nailing of the femur. The area of the nonunion is localized with intraoperative fluoroscopy. If not already done so, any intramedullary nail or other obstructing implants around the nonunion are removed. A 1 cm incision is made at the level of the nonunion just lateral to the tibial crest (for tibial nonunions) or through the lateral aspect of the thigh’s anterior compartment (for femoral nonunions). Blunt dissection is performed through the incision to gently mobilize the soft tissue from the underlying bone. We use a 3.5 mm drill bit to drill multiple transcortical vents around the nonunion site through the 1 cm incision. Additional 1 cm incisions are made as needed to ensure drill bit access throughout the nonunion site. Care is taken to avoid thermal injury while drilling the cortical vents by operating the drill at low speed, stopping frequently, and irrigating the drill bit with cool water. A standard exchange nailing is then performed in which the intramedullary canal is over-reamed and a larger diameter nail is inserted and statically locked within the canal. We allow our patients to bear full weight on the operative extremity following the surgery to further encourage union.

## Case presentation

The following consecutive series of cases treated by the senior author highlight this adjunctive technique’s potential effectiveness in helping to achieve successful bone healing when treating high-risk or recalcitrant nonunions (Table 1).

**Table 1:** Case series injury, patient, and treatment characteristics *: AO/OTA Fracture Classification; I&D: irrigation and debridement

Patient	Initial injury^*^	Age at injury (years)	Risk factor for nonunion	Type of nonunion	Additional procedures	Definitive nonunion treatment performed in conjunction with cortical venting technique	Length of follow-up after definitive nonunion treatment
1	42B2	30	Comminution, compartment syndrome	Oligotrophic	Exchange nailing x2 with bone grafting	Staged exchange nailing	48 months
2	42A3	69	Smoker, advanced age, open fracture, osteomyelitis	Hypertrophic	Removal of hardware (nail), application of Ilizarov external fixator frame, I&D (osteomyelitis)	Motorized telescoping intramedullary nail compressing across the nonunion	48 months
3	42B2	56	Smoker, open segmental fracture requiring flap coverage, multiple surgeries	Oligotrophic	Exchange nailing	Staged exchange nailing	60 months
4	33A3	32	Open comminuted fracture, need for skin graft coverage, polytrauma	Oligotrophic	Retrograde IMN, revision to distal femur lateral locking plate with bone grafting, removal of distal femur lateral locking plate	Single-stage exchange nailing	72 months

Case 1

A 30-year-old male non-smoker sustained a closed, comminuted tibial shaft fracture (AO/OTA 42B2), a segmental fibula fracture, and a syndesmotic injury of his left lower extremity resulting from a motorcycle crash. Initial treatment of these fractures performed at another institution consisted of intramedullary nailing of the tibia and the distal fibula and fixation of the syndesmosis with a syndesmotic screw. His immediate postoperative course was complicated by the onset of compartment syndrome of the leg, necessitating a four-compartment fasciotomy. The patient subsequently developed a tibial nonunion for which he underwent two exchange nailing procedures with open bone grafting over the ensuing two years.

**Figure 1:**
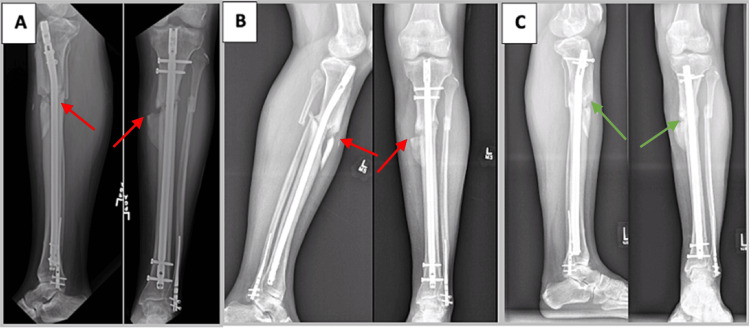
A 30-year-old man with oligotrophic tibial nonunion (case 1) A) Radiographs of the patient’s left leg taken eight months after injury demonstrating a nonunited (red arrows) and deformed tibia. B) Persistent nonunion (red arrows) 12 months following the patient’s second exchange nailing and allogeneic bone grafting. At this time, priority was placed on achieving union through a third exchange nailing rather than concomitantly addressing the tibia’s residual deformity. C) Tibial union (green arrows) is evident on radiographs taken one year after performing our modified exchange nailing operation utilizing cortical vent tunnels. The patient has since declined to proceed with tibial malunion correction surgery.

Despite these two exchange nailing procedures, the patient’s nonunion persisted at which time he was referred to the senior author for potential transtibial amputation (Figures 1A, 1B). Radiographs obtained at the time of this presentation demonstrated a nonunited tibia that was shortened, in valgus, and externally rotated. Given the patient’s frustration and willingness to consider amputation, the treatment strategy was tailored to treat his nonunion first with a staged exchange nailing and then address his tibia deformity at a later date. During the first stage of treating the nonunion, the intramedullary nail was removed and intramedullary cultures were obtained. Upon ruling out infection with the finalization of the cultures, the second stage was performed in which percutaneous cortical vent tunnels were drilled in conjunction with a third exchange nailing. Within a few months of this third exchange nailing operation, the patient reported decreased pain, and his radiographs demonstrated callus formation at the nonunion site. Radiographs obtained 12 months following this revision demonstrated tibial union, by which time he had returned to running and other recreational athletic activities (Figure 1C). Since then, he decided to forgo subsequent deformity correction surgery.

Case 2

**Figure 2:**
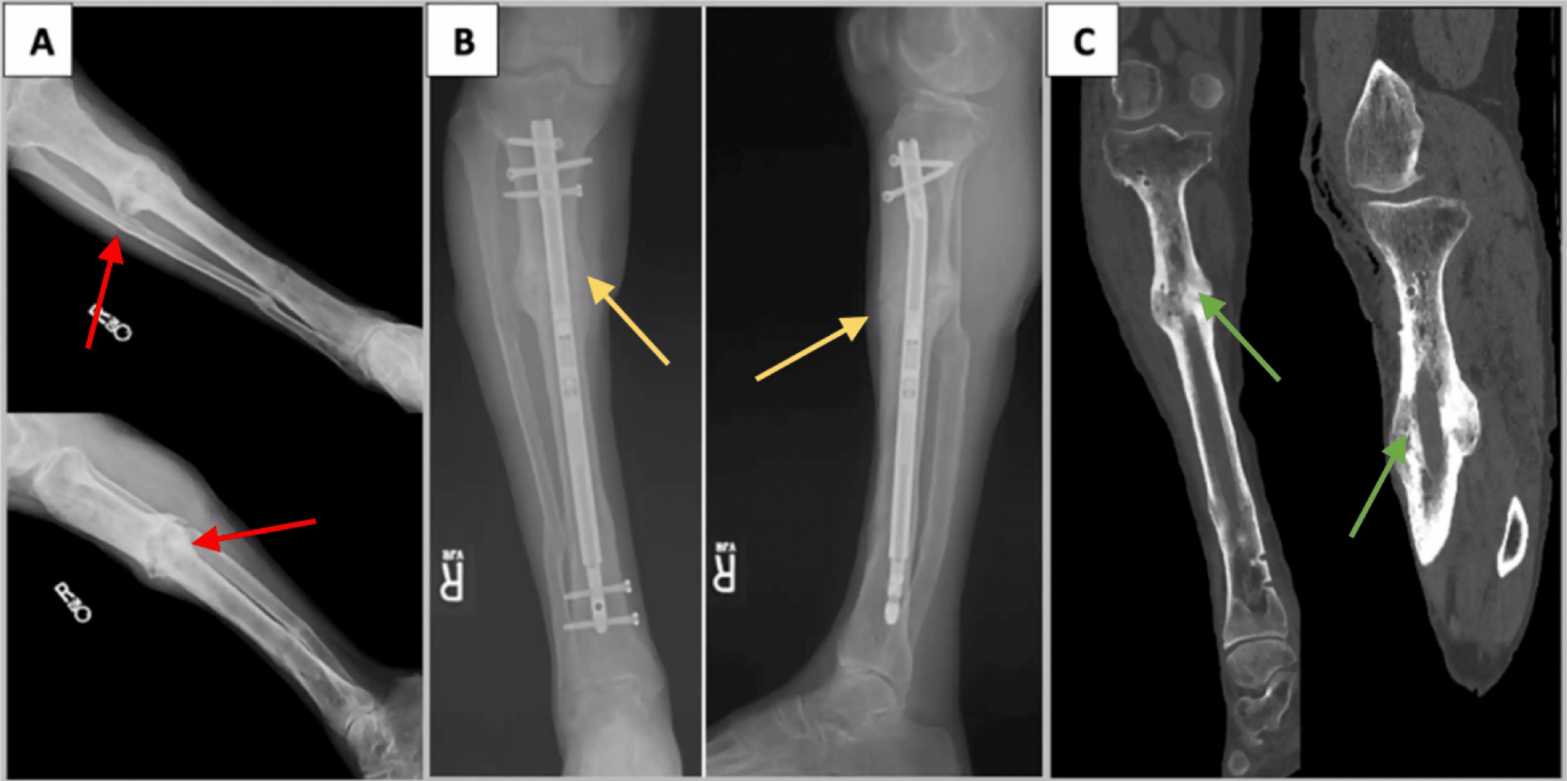
A 69-year-old male smoker with a hypertrophic tibial nonunion (case 2) A) Radiographs after failed Ilizarov external fixation treatment complicated by osteomyelitis demonstrating a persistent hypertrophic nonunion (red arrows). B) Approximately one year after exchange nailing with cortical venting and insertion of a telescoping motorized intramedullary nail in which bridging callous formation (yellow arrows) is evident. C) CT of the tibia at 18 months after modified exchanged nailing procedure and subsequent intramedullary nail removal demonstrating union (green arrows).

A 69-year-old male smoker developed a nonunion following intramedullary nailing of an open tibial shaft fracture (AO/OTA 42A3) resulting from a forklift accident. Prior to the presentation, he underwent several nonunion surgeries over an 11-year period, starting with an exchange nailing with open bone grafting approximately one year after injury. Seven years later, he underwent removal of the intramedullary nail for presumed symptomatic hardware that unbeknownst at that time was actually a hypertrophic nonunion (Figure 2A). Two years later, he underwent Ilizarov ringed external fixation treatment for his nonunion. Unfortunately, the patient developed osteomyelitis of the tibia during the course of this treatment requiring irrigation and debridement and intravenous antibiotics to eradicate the infection. His nonunion persisted following a year of Ilizarov treatment so further treatment with the ringed external fixator was discontinued at that time.

Approximately one year following the removal of the external fixator, treatment of the tibial nonunion was reinitiated with the insertion of a motorized telescoping intramedullary nail used to compress across the nonunion. Prior to reaming the canal, cortical vents were percutaneously drilled using the described technique. The nail was incrementally compressed by 2 mm every four weeks throughout the postoperative period. The patient’s pain continued to improve as his follow-up radiographs demonstrated progressive union over the ensuing months. One year following this surgery, radiographs showed a united tibia and the patient’s pain had resolved (Figure 2B). The nail was removed 18 months after placement, and the patient remained pain- and complication-free since then (Figure 2C).

Case 3

**Figure 3:**
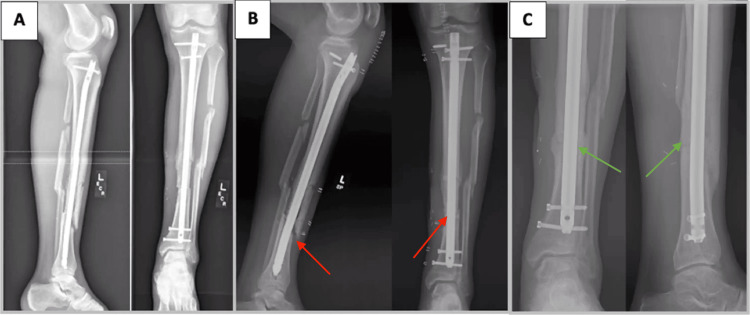
A 56-year-old male smoker with oligotrophic nonunion of a segmental tibial fracture (case 3) A) Radiographs of the patient’s left tibia four months after injury, status post tibia and fibula intramedullary nailing at an outside hospital with soleus flap coverage and skin grafting. B) Radiographs of the left tibia demonstrating persistent nonunion (red arrows) with no callous formation at fracture site six months after index surgery. C) Radiographs demonstrating union (green arrows) approximately one year after exchange nailing performed with adjunctive cortical vent drill tunnels. The patient was pain-free at the former tibial nonunion site.

A 56-year-old male smoker sustained an open tibial shaft fracture (AO/OTA 42B2) resulting from a motorcycle crash. This injury was treated with debridement of the wound, intramedullary nailing of the tibia and fibula, a soleus flap, and split-thickness skin grafting (Figure 3A). The patient subsequently developed an aseptic nonunion of the tibia fracture (Figure 3B). Six months after the index surgery, he underwent staged exchange nailing. In the first stage, the initial nail was removed and tissue cultures were obtained. He underwent an exchange nailing two weeks later with percutaneously drilled cortical vents after confirming that his nonunion was aseptic. Over the next several months, the patient reported improvement in his pain as the nonunion healed. Although the patient achieved bony union following this surgery, he was unable to progress to running due to ipsilateral symptomatic ankle osteoarthritis (Figure 3C).

Case 4

**Figure 4:**
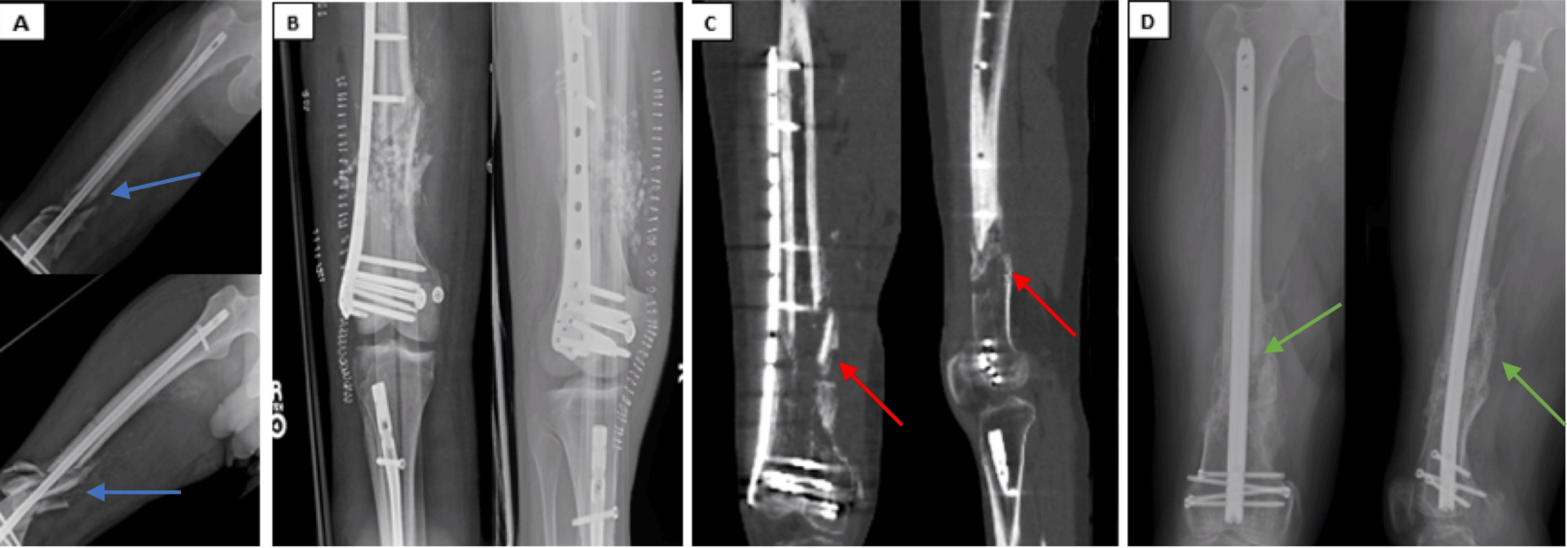
A 32-year-old man with an open, comminuted distal femur fracture that developed into an oligotrophic nonunion (case 4) A) Radiographs four weeks status post retrograde nailing for the comminuted distal third femur fracture (blue arrows) at an outside institution, with approximately two centimeters of shortening and no evidence of callus formation at the fracture site. Ipsilateral tibial nailing was performed at the time of initial retrograde femoral nailing for an open tibia fracture. B) Immediate postoperative imaging status post femoral nail removal with revision to lateral distal femur locking plate and bone grafting. C) CT scan eight months after femoral plating and bone grafting operation demonstrating a persistent nonunion (red arrows). D) Radiographs nine months after exchange nailing with pre-drilled cortical vent tunnels demonstrating robust bridging bone formation (green arrows) at the former nonunion site.

A 32-year-old male sustained a right open comminuted distal third diaphyseal femur fracture (AO/OTA 33A3) and several other lower extremity and pelvic fractures in a motorcycle crash. He initially underwent external fixation of the femur fracture and skin grafting over the medial thigh wound before undergoing a right femur retrograde intramedullary nailing at an outside institution. He subsequently presented to our institution with his quadriceps tendon in discontinuity, transected by the butterfly fragment approximately four weeks after his injury. At that point, no callus was noted around the intramedullary implant (Figure 4A). He was revised to a lateral distal femur locking plate with bone grafting and quadriceps tendon repair after infection was ruled out (Figure 4B). He subsequently underwent additional surgeries on his contralateral lower extremity, which resulted in increased functional demands on the healing right femur fracture due to contralateral weight-bearing restrictions. He continued to experience right thigh pain eight months after his femur plating, at which time a CT demonstrated a persistent oligotrophic femoral nonunion with 2.5 cm of shortening (Figure 4C). At this point, the patient underwent the removal of hardware and retrograde intramedullary nailing performed in conjunction with pre-drilling cortical vents around the nonunion. The patient went on to achieve union with robust bridging bone over the ensuing months (Figure 4D). The patient resumed full weight bearing on the right lower extremity without any complications related to the former femoral nonunion; however, he continued to experience considerable functional challenges due to his contralateral lower extremity injuries. As a result, he elected to undergo contralateral transtibial amputation two years after his initial injury, but he continued to have reasonable weight-bearing function with his right lower extremity.

## Discussion

The described cortical venting technique adapts the use of percutaneous drill osteotomies often utilized in a motorized telescoping intramedullary nail limb lengthening procedure to the exchange nailing procedure commonly performed in long bone nonunion surgery. In the limb lengthening setting, a series of percutaneous holes are drilled at the planned osteotomy site, followed by reaming of the intramedullary canal and completion of the osteotomy site [16,17]. Proceeding in this sequence allows for the local accumulation of vented osteogenic reamings, which then enhances the development of regenerate bone formation [17].

While the local deposition of osteogenic intramedullary reamings is assured in fresh fractures, the fracture site of most nonunions is filled with fibrous tissue. We contend that this fibrous tissue prevents most, if not all, intramedullary reamings generated during an exchange nailing from being deposited at the nonunion site, in contrast to the local extrusion of reamings that occurs while reaming a fresh fracture. Instead, the fibrous tissue directs these reamings away from the nonunion site either into the distal extent of the canal or causes them to be extruded proximally through the entry portal when performing an exchange nailing. These cortical vents allow for egress of osteogenic intramedullary reamings into and around the nonunion, thereby positioning the autograft at the nonunion site and encouraging new bridging bone to form there.

Drilling the cortical vents recruits osteoinductive mediators by stimulating bleeding in the area. These osteoinductive mediators induce the extruded reamings’ mesenchymal cells to differentiate into chondrocytes. In turn, these chondrocytes secrete cartilage matrix before undergoing hypertrophic differentiation that mineralizes the surrounding matrix, resulting in bone callus formation [18]. We contend that this optimized biologic environment around the nonunion site complements the effect of increased mechanical stability associated with insertion of a larger intramedullary nail in the exchange nailing and therefore further enhances the nonunion’s ability to heal.

There are several advantages to the described adjunctive cortical venting technique. We contend that it is simple and quick to perform, utilizing instruments already available for performing an exchange nailing procedure. Moreover, the relatively small incisions made to accommodate percutaneous drilling enable the accumulation of bone autograft around the nonunion while bypassing the inherent morbidity of making an open approach to the area for applying bone graft. This is particularly beneficial in patients with a compromised soft tissue envelope surrounding the nonunion.

We achieved successful outcomes using the adjunctive cortical venting technique in our consecutive series of recalcitrant nonunions presented. However, we acknowledge that more definitive validation of its effectiveness requires a much larger patient cohort in which the technique is utilized. Furthermore, a larger and more diverse cohort will increase the generalizability of this technique in addition to potentially providing more insights into the effects of cortical venting on healing a nonunion. Other factors that need to be further elucidated through further investigation of the technique are determining the ideal drill bit size to be used and the number of drill osteotomies created. Hypothetically, the larger the drill bit used and the higher the number of vents created will lead to more osteogenic reamings exiting the canal and surrounding the nonunion site. We use a 3.5 mm drill bit because we consider this to be of adequate size for allowing extrusion of the intramedullary reamings without substantially raising the stress riser-induced fracture risk during reaming of the canal and nail insertion. In addition, further investigation should focus on better quantifying the amount of bone graft generated with this technique.

## Conclusions

Even with the multitude of treatment options available, long bone nonunion remains a challenging problem for orthopedic surgeons. Our success with the described cortical venting technique performed with the exchange nailing procedure highlights its potential benefit by facilitating the dispersion of osteogenic intramedullary reamings around the nonunion site. This adjunctive technique is simple, quick, and cost-effective. Further study is needed to gain a more comprehensive understanding of its effectiveness in healing a nonunion.
